# Complete genome of the extensively antibiotic-resistant GC1 *Acinetobacter baumannii* isolate MRSN 56 reveals a novel route to fluoroquinolone resistance

**DOI:** 10.1093/jac/dkac115

**Published:** 2022-04-11

**Authors:** Christopher J Harmer, Francois Lebreton, Jason Stam, Patrick T McGann, Ruth M Hall

**Affiliations:** School of Life and Environmental Sciences, The University of Sydney, Sydney, Australia; Multidrug Resistant Organism Repository and Surveillance Network, Walter Reed Army Institute of Research, Silver Spring, MD, USA; Multidrug Resistant Organism Repository and Surveillance Network, Walter Reed Army Institute of Research, Silver Spring, MD, USA; Multidrug Resistant Organism Repository and Surveillance Network, Walter Reed Army Institute of Research, Silver Spring, MD, USA; School of Life and Environmental Sciences, The University of Sydney, Sydney, Australia

## Abstract

**Objectives:**

To examine the causes of antibiotic resistance in the extensively resistant global clone 1 (GC1) *Acinetobacter baumannii* isolate MRSN 56 recovered at a US military treatment facility.

**Methods:**

MRSN 56 was sequenced using MinION (Oxford Nanopore) and the reads combined with available Illumina MiSeq data using Unicycler. Acquired resistance genes were identified using ABRicate and their environment examined. ISAba1 and ISAba125 copies were located.

**Results:**

MRSN 56 is ST1^IP^:ST231^Ox^:KL1:OCL1 and the complete genome includes four small plasmids, none of which carry resistance genes. The acquired resistance genes were found at four locations in the chromosome in addition to AbaR28 (*aphA1*, *aacC1*, *aadA1*, *sul1*) in *comM*. Tn*2006* (*oxa23*, carbapenem resistance) was both in AbaR4 and alone elsewhere. Two copies of Tn*7* (*dfrA1*, *sat*, *aadA1*) were identified. One was associated with a 22* *852 bp adjacent segment [*tetA*(B), *sul2*] derived from the AbGRI1 island, and this novel configuration was designated Tn*7+*. Tn*7+* was incorporated in the position preferred by Tn*7*, downstream of *glmS*, by transposition using a sequence in AbGRI1 resembling the Tn*7* terminal inverted repeats. Tn*7* was found at a secondary site. Fluoroquinolone resistance appears to involve a mutation in *gyrA* combined with inactivation by ISAba1 of the *marR* gene in the *mar* operon and constitutive expression of *marA* from the promoter internal to ISAba1.

**Conclusions:**

MRSN 56 represents a new sublineage of GC1 lineage 1 with novel features that had not been detected previously. The involvement of the *mar* operon in fluoroquinolone resistance has not been noted previously.

## Introduction

In the context of development of extensive- or pan-antibiotic resistance, the contribution of the *Acinetobacter baumannii* clonal complex 1 (CC1), also known as global clone 1 (GC1), is understudied and its distribution and diversity are underappreciated. A detailed bioinformatic analysis of available sequences revealed two main lineages.^1^ Lineage 1 emerged around 1960 and the founding event was the acquisition of the AbaR0 resistance island in *comM.* Lineage 1 divided into the AbaR0- and AbaR3-derived branches^2,3^ around 1980. Various sublineages were distinguished by the mode of development of resistance to antibiotics introduced later, fluoroquinolones, third-generation cephalosporins and carbapenems.^1^ A later study of one sublineage of the AbaR3 branch revealed that its history could be traced via the location of transposons and ISs.^4^

MRSN 56 is an extensively resistant GC1/CC1 *A. baumannii* isolate recovered in 2010 from the wounds of a 20-year-old soldier injured in Afghanistan and returned to the USA for treatment.^5^ Treatment with tobramycin, one of the few remaining options, led to tobramycin resistance arising from the substantial amplification of Tn*6020*, which carries the *aphA1* gene.^5^ However, only short-read data were available for MRSN 56 and this limits the ability to identify chromosomal alterations leading to antibiotic resistance and to examine the genomic context of the acquired resistance genes. Hence, the complete genome of this isolate was determined and the location of each of the acquired resistance genes and the chromosomal changes leading to resistance were examined.

## Methods

### Genome sequencing and annotation

The complete genome of MRSN 56 was determined by combining newly determined MinION long-read data (Oxford Nanopore) and available short-read Illumina Miseq data (SRR14998418). A hybrid assembly was generated using Unicycler as described elsewhere.^6,7^ Briefly, the MinION reads were filtered using Fitlong version 0.2.1 (https://github.com/rrwick/Filtlong) to remove reads <1000 bp and 500 Mb of long reads were combined with the short-read data using Unicycler version 0.4.0 with default settings.^8^ The copy number of plasmids was determined by comparing the coverage of the plasmid to that of the chromosome (copy number = 1) using an average of the coverage of Institut Pasteur MLST alleles.

Protein coding, rRNA and tRNA genes were initially annotated using a combination of Prokka (version 1.12)^9^ and RAST.^10^ Antibiotic resistance genes were identified using ABRicate version 0.8.13 (https://github.com/tseemann/abricate) and plasmids and resistance regions were annotated manually using an in-house database of standard sequences. The PubMLST database (www.pubmlst.org) was used to determine STs. Kaptive^11^ was used to identify surface polysaccharide types and the KL and OCL loci were annotated manually. The *ampC* database^12^ in PubMLST was used to identify *ampC* alleles.

### Resistance and analysis of resistance regions

Resistance to antibiotics was determined as described previously.^13^ Antibiotic resistance regions were identified and analysed using an in-house database of known transposons and resistance islands with stand-alone BLAST. Regions that did not match a known structure in our database were used as queries in BLASTn searches of the GenBank nucleotide database.

### GenBank accession numbers

The sequences of the MRSN 56 chromosome and four plasmids, pMRSN56-1, pMRSN56-2, pMRSN56-3 and pMRSN56-4, have been deposited in GenBank (BioProject PRJNA742487) under accession numbers CP080452, CP080453, CP080454, CP080455 and CP080456, respectively. Reads are available under accession numbers SRR14998418 (Illumina MiSeq) and SRR14008417 (Oxford Nanopore).

## Results and discussion

### Resistance profile of MRSN 56

Among the antibiotics tested, MRSN 56 was recorded as resistant to ampicillin, ampicillin/sulbactam, aztreonam, cefazolin, the carbapenems imipenem and meropenem, and the third- and fourth-generation cephalosporins ceftazidime, ceftriaxone and cefepime.^5^ It was also resistant to the fluoroquinolones ciprofloxacin, levofloxacin and moxifloxacin, and the aminoglycoside gentamicin, as well as to trimethoprim/sulphamethoxazole, tetracycline and nitrofurantoin. MRSN 56 was susceptible only to tigecycline, amikacin and tobramycin and to colistin (MIC < 0.25 mg/L).

### Complete genome of MRSN 56

The complete genome of MRSN 56 assembled from newly determined long-read data and available short-read data includes the chromosome and four small plasmids (Table 1). MRSN 56 belongs to ST1 in the Institut Pasteur scheme^5^ and to ST231 in the Oxford scheme, making it a member of GC1. The chromosome carries the GC1-associated variant of the intrinsic *oxaAb* gene encoding the OXA-69 variant, and includes the KL1 gene cluster at the capsule locus and OCL1 at the variable outer core locus, a configuration seen in several other GC1/CC1 isolates.^1,13^

**Table 1.** MRSN 56 genome content

Replicon	Size (bp)	Copy number	Replicon type	Accession number	Comments
Chromosome	4* *033* *258	1	—	CP080452	
pMRSN56-1	2178	4	Rep_1	CP080453	Novel Rep
pMRSN56-2	2725	3	Rep_1	CP080454	Identical to pA85-1^a^
pMRSN56-3	6772	3–4	Rep_3	CP080455	Novel Rep
pMRSN56-4	8731	3	Rep_3	CP080456	Identical to pA1-1^b^, RepAci1

a GenBank accession number CP021783.

b GenBank accession number CP010782.

**Figure 1.**
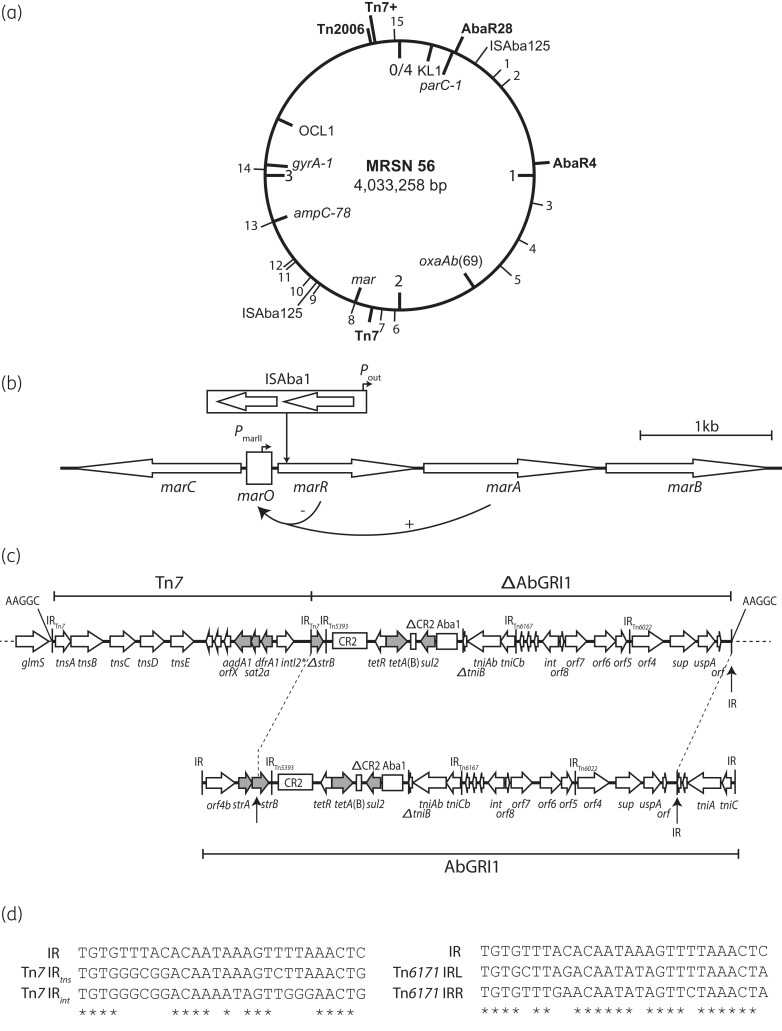
Features of MRSN 56. (a) Circular map of the chromosome of *A. baumannii* strain MRSN 56. Locations of important features, e.g. *gyrA*, *parC*, *oxaAb*, *mar* and *ampC* genes, KL and OCL, and the location of insertions (e.g. AbaR, Tn*2006*, Tn*7*, Tn*7+*) are indicated. Thin black numbered lines indicate the locations of solo ISAba1 copies. Further copies surround Tn*2006* (the solo copy shown and the copy in AbaR4) and Tn7+ includes a copy. Drawn to scale from GenBank accession number CP080452 (MRSN 56). (b) Structure of the *mar* operon showing the location and orientation of ISAba1 copy 8. (c) Structure of Tn*7*+. The chromosome on each side of the island is represented by a thin dashed line. ISs are shown as boxes, and inverted repeats (IRs) are shown as vertical bars. The nucleotide sequence of target site duplications is indicated by letters above. The orientation and extent of genes are indicated by horizontal arrows. Structures of known origin are labelled. Drawn to scale from GenBank accession numbers CP080452 and JN968483. (d) Comparison of the TIRs of Tn*7* and Tn*6167* with the novel TIR found at the right boundary of Tn*7*+.

Twenty copies of ISAba1 were detected in the MRSN 56 chromosome. Five are part of the resistance regions described below, four at the boundaries of Tn*2006* and one in Tn*7*+. The remaining 15, including one upstream of *ampC*, are located throughout the chromosome as shown in Figure 1a. Two copies of ISAba125 were also present.

### Acquired resistance involving intrinsic genes

The copy of ISAba1 found upstream of the intrinsic *ampC* gene in MRSN 56 would lead to cephalosporin resistance. The *ampC* allele was compared with that found in early GC1 isolates such as A1^14^ (GenBank accession number CP010781) representing the standard GC1 sequence, and a recombination patch internal to *ampC* with 28 single-base substitutions between bases 77 and 1044 was detected. This allele is classified as *ampC-*78.^12^ No IS was present upstream of the intrinsic chromosomal *oxaAb* gene encoding OXA-69 and this allele is not known to be associated with reduced susceptibility to carbapenems.^15^

Fluoroquinolone resistance could not be explained by the presence of codon alterations at known positions in the resistance-determining regions of *gyrA* and *parC*, as only the S81L substitution in *gyrA*, which confers resistance to nalidixic acid, was detected. Inspection of the *gyrB*, *parC* and *parA* genes revealed no substitution in GyrB, ParC or ParA relative to the standard GC1 alleles seen in the chromosome of the early fluoroquinolone-susceptible isolate A1. As we were unable to find the source of the fluoroquinolone resistance at these locations, we searched among the genes that would be expressed from the strong outward-facing promoter in an ISAba1 located near them. The ISAba1 numbered 8 in Figure 1a was found to be located in a gene encoding a protein that is 37% identical to the MarR repressor of the *marAB* genes of *Escherichia coli.* In MRSN 56, the *marR* gene is followed by genes encoding MarA and MarB proteins that are 32% and 29% identical, respectively, to the well-studied Mar proteins of *E. coli* and the ISAba1 is oriented such that the *marA* and *marB* genes would be constitutively expressed from its internal promoter (Figure 1b). In *E. coli*, it has been shown that expression of MarA, a transcriptional activator, increases expression of a set of genes that lead to multiple antibiotic resistance.^16,17^ In addition, expression of MarA has been shown to reduce susceptibility to norfloxacin^17^ and it is likely that the inactivation of the *marR* gene plus provision of a replacement promoter internal to the ISAba1 combined with the S81L substitution in GyrA accounts for the observed fluoroquinolone resistance.

### Context of acquired antibiotic resistance genes in MRSN 56

The acquired antibiotic resistance genes in the MRSN 56 genome were all included in the chromosome but were distributed among five different transposon insertions (or islands). As reported previously,^5^ MRSN 56 retains only a remnant of the AbaR island typically found in *comM* in GC1 isolates^3^ and the IS*26* at one end of Tn*6020* in AbaR28 has caused a deletion (see Figure 1b in^18^) that extends for 55.7 kb into the adjacent chromosomal sequence.^5^ AbaR28 retains the *aphA1b* gene in Tn*6020*^19^ and the *aacC1*, *aadA1* and *sul1* genes of the class 1 integron. The small (108 bp) deletion in *intI1* that distinguishes the two main groups in GC1 lineage 1 that carry an AbaR derived from AbaR0 (*intI1* intact) or from AbaR3 (*intI1* deletion)^1,2^ was present. The AbaR3 branch of GC1 lineage 1 is estimated to have arisen around 1985.^1^ The *oxa23* carbapenem resistance gene is in Tn*2006*, which is present twice, once located in AbaR4 and once alone (target site duplication agcatagat). The second copy of Tn*2006* may be derived from AbaR4.

Two copies of Tn*7*, carrying the *dfrA1* trimethoprim resistance gene, the *sat2* streptothricin resistance gene and another copy of *aadA1*, were identified. One copy, part of Tn*7*+, was found in the preferred location for Tn*7*, namely downstream of *glmS*, and the second was in a secondary site (see Figure 1a for locations of Tn*7*+ and Tn*7*). However, the copy near *glmS* was associated with an adjacent segment and together they were surrounded by the expected 5 bp target site duplication, suggesting that they had moved to this location together. The additional segment included the *sul2* sulphonamide resistance gene and the *tet*(B) tetracycline resistance determinant (Figure 1c) and the presence of *tet*(B) explains the observed tetracycline resistance. The combined structure was designated Tn*7* +.

### Origin of Tn7+

The segment adjacent to Tn*7* is identical to part of the backbone of the full-length AbGRI1^20^ and the configuration found on plasmid pAB3 from ATCC 17978 from which AbGRI1 originated.^21^ To explain how this segment could have moved together with Tn*7*, the sequence at the outer boundary with the chromosome was examined and a sequence related to the inner transposase-binding region of the Tn*7* terminal inverted repeat (TIR) was detected (Figure 1d). This sequence is more closely related to the TIR of Tn*6171*, a transposon related to Tn*7* that includes the fimsbactin synthesis and uptake genes and also targets *att*Tn*7*.^22^ However, the presence of this sequence in the Tn*6022* portion of AbGRI1 appears to be fortuitous.

The proposed route to generation of Tn*7+* is as follows. First, Tn*7* jumped into pAB3 or AbGRI1 at the position in *strB* marked by the vertical arrow in Figure 1c. Then it was cut out from this position by the Tn*7* transposition machinery using the fortuitous TIR-like sequence at one end (IR in Figure 1c) and this combination was pasted into the standard *att*Tn*7* location downstream of *glmS*.

### Conclusions

MRSN 56 represents a new sublineage of GC1 that was not detected in a previous detailed analysis of sequenced GC1 isolates.^1^ Unusual chromosomal modifications and the locations of acquired antibiotic resistance regions (e.g. AbaR4, Tn*7*) and ISAba1 copies should simplify identification of close relatives among complete and draft genomes. The fluoroquinolone resistance appears to have arisen via an unusual route but further work will be needed to confirm the role of MarR inactivation in fluoroquinolone resistance.

## References

[1] Holt K , Kenyon JJ, Hamidian M et al Five decades of genome evolution in the globally distributed, extensively antibiotic-resistant *Acinetobacter baumannii* global clone 1. Microb Genom 2016; 2: e000052. 28348844 10.1099/mgen.0.000052 PMC5320584

[2] Hamidian M , Wynn M, Holt KE et al Identification of a marker for two lineages within the GC1 clone of *Acinetobacter baumannii*. J Antimicrob Chemother 2014; 69: 557–8. 24080502 10.1093/jac/dkt379 PMC3886933

[3] Hamidian M , Hall RM. The AbaR antibiotic resistance islands found in *Acinetobacter baumannii* global clone 1 - structure, origin and evolution. Drug Resist Updat 2018; 41: 26–39. 30472242 10.1016/j.drup.2018.10.003

[4] Hamidian M , Hawkey J, Wick R et al Evolution of a clade of *Acinetobacter baumannii* global clone 1, lineage 1 via acquisition of carbapenem- and aminoglycoside-resistance genes and dispersion of ISAba1. Microb Genom 2019; 5: e000242. 10.1099/mgen.0.000242 PMC6412058 30648939

[5] McGann P , Courvalin P, Snesrud E et al Amplification of aminoglycoside resistance gene *aphA1* in *Acinetobacter baumannii* results in tobramycin therapy failure. mBio 2014; 5: e00915. 10.1128/mBio.00915-14 PMC3994513 24757213

[6] Harmer CJ . HI1 and I1 resistance plasmids from *Salmonella enterica* serovar Typhimurium strain SRC27 are epidemic. Microb Drug Resist 2021; 27: 1495–504. 34242087 10.1089/mdr.2020.0579

[7] Hamidian M , Wick RR, Judd LM et al Complete genome sequence of A388, an antibiotic-resistant *Acinetobacter baumannii* global clone 1 isolate from Greece. Microbiol Resour Announc 2019; 8: e00971-19. 31601668 10.1128/MRA.00971-19 PMC6787325

[8] Wick RR , Judd LM, Gorrie CL et al Unicycler: resolving bacterial genome assemblies from short and long sequencing reads. PLoS Comput Biol 2017; 13: e1005595. 28594827 10.1371/journal.pcbi.1005595 PMC5481147

[9] Seemann T . Prokka: rapid prokaryotic genome annotation. Bioinformatics 2014; 30: 2068–9. 24642063 10.1093/bioinformatics/btu153

[10] Aziz RK , Bartels D, Best AA et al The RAST Server: rapid annotations using subsystems technology. BMC Genomics 2008; 9: 75. 18261238 10.1186/1471-2164-9-75 PMC2265698

[11] Wyres KL , Cahill SM, Holt KE et al Identification of *Acinetobacter baumannii* loci for capsular polysaccharide (KL) and lipooligosaccharide outer core (OCL) synthesis in genome assemblies using curated reference databases compatible with Kaptive. Microb Genom 2020; 6: e000339. 10.1099/mgen.0.000339 PMC7200062 32118530

[12] Karah N , Jolley KA, Hall RM et al Database for the *ampC* alleles in *Acinetobacter baumannii*. PLoS One 2017; 12: e0176695. 28459877 10.1371/journal.pone.0176695 PMC5411055

[13] Galac MR , Snesrud E, Lebreton F et al A diverse panel of clinical *Acinetobacter baumannii* for research and development. Antimicrob Agents Chemother 2020; 64: e00840-20. 32718956 10.1128/AAC.00840-20 PMC7508605

[14] Holt KE , Hamidian M, Kenyon JJ et al Genome sequence of *Acinetobacter baumannii* strain A1, an early example of antibiotic-resistant global clone 1. Genome Announc 2015; 3: e00032-15. 25767221 10.1128/genomeA.00032-15 PMC4357743

[15] Takebayashi Y , Findlay J, Heesom KJ et al Variability in carbapenemase activity of intrinsic OxaAb (OXA-51-like) β-lactamase enzymes in *Acinetobacter baumannii*. J Antimicrob Chemother 2021; 76: 587–95. 33338207 10.1093/jac/dkaa502

[16] Alekshun MN , Levy SB. Regulation of chromosomally mediated multiple antibiotic resistance: the *mar* regulon. Antimicrob Agents Chemother 1997; 41: 2067–75. 9333027 10.1128/aac.41.10.2067 PMC164072

[17] Alekshun MN , Levy SB. The *mar* regulon: multiple resistance to antibiotics and other toxic chemicals. Trends Microbiol 1999; 7: 410–3. 10498949 10.1016/s0966-842x(99)01589-9

[18] Harmer CJ , Hall RM. IS*26*-mediated formation of transposons carrying antibiotic resistance genes. mSphere 2016; 1: e00038-16. 10.1128/mSphere.00038-16 PMC4894685 27303727

[19] Post V , Hall RM. AbaR5, a large multiple-antibiotic resistance region found in *Acinetobacter baumannii*. Antimicrob Agents Chemother 2009; 53: 2667–71. 19364869 10.1128/AAC.01407-08 PMC2687260

[20] Nigro SJ , Brown MH, Hall RM. AbGRI1-5, a novel AbGRI1 variant in an *Acinetobacter baumannii* GC2 isolate from Adelaide, Australia. J Antimicrob Chemother 2019; 74: 821–3. 30452642 10.1093/jac/dky459

[21] Hamidian M , Hall RM. Origin of the AbGRI1 antibiotic resistance island found in the *comM* gene of *Acinetobacter baumannii* GC2 isolates. J Antimicrob Chemother 2017; 72: 2944–7. 28666372 10.1093/jac/dkx206

[22] Hamidian M , Hall RM. Dissemination of novel Tn*7* family transposons carrying genes for synthesis and uptake of fimsbactin siderophores among *Acinetobacter baumannii* isolates. Microb Genom 2021; 7: e000548. 10.1099/mgen.0.000548 PMC8190619 33749577

